# Comparative transcriptomic analysis of PK15 cells infected with a PRV variant and the Bartha-K/61 vaccine strain

**DOI:** 10.3389/fmicb.2023.1164170

**Published:** 2023-05-05

**Authors:** Hongliang Zhang, Xiaoxiao Duan, Gang Liu, Yingguang Li, Shaoming Dong, Jiaxu Lin, Ruihua Zhang, Xiulei Cai, Hu Shan

**Affiliations:** ^1^Shandong Collaborative Innovation Center for Development of Veterinary Pharmaceuticals, College of Veterinary Medicine, Qingdao Agricultural University, Qingdao, China; ^2^Qingdao Animal Disease Prevention and Control Center, Qingdao, China; ^3^Key Laboratory of Preventive Veterinary Medicine, Department of Veterinary Medicine, Animal Science College, Hebei North University, Zhangjiakou, China

**Keywords:** pseudorabies virus, mutant, Bartha-K/61, PK15 cells, RNA-seq, transcriptomic

## Abstract

**Introduction:**

Pseudorabies virus (PRV) is a herpesvirus that can infect domestic animals, such as pigs, cattle and sheep, and cause fever, itching (except pigs), and encephalomyelitis. In particular, the emergence of PRV variants in 2011 have resulted in serious economic losses to the Chinese pig industry. However, the signaling pathways mediated by PRV variants and their related mechanisms are not fully understood.

**Methods:**

Here, we performed RNA-seq to compare the gene expression profiling between PRV virulent SD2017-infected PK15 cells and Bartha-K/61-infected PK15 cells.

**Results:**

The results showed that 5,030 genes had significantly different expression levels, with 2,239 upregulated and 2,791 downregulated. GO enrichment analysis showed that SD2017 significantly up-regulated differentially expressed genes (DEGs) were mainly enriched in the binding of cell cycle, protein and chromatin, while down-regulated DEGs were mainly enriched in ribosomes. KEGG enrichment analysis revealed that the pathways most enriched for upregulated DEGs were pathways in cancer, cell cycle, microRNAs in cancer, mTOR signaling pathway and autophagy-animal. The most down-regulated pathways of DEGs enrichment were ribosome, oxidative phosphorylation, and thermogenesis. These KEGG pathways were involved in cell cycle, signal transduction, autophagy, and virus-host cell interactions.

**Discussion:**

Our study provides a general overview of host cell responses to PRV virulent infection and lays a foundation for further study of the infection mechanism of PRV variant strain.

## Introduction

Pseudorabies (PR), also known as Aujeszky's disease (AD), is an acute and severe infectious disease caused by Pseudorabies virus (PRV) (Wozniakowski and Samorek-Salamonowicz, 2015). PRV can infect a variety of mammals, including humans, pigs, dogs, and rodents (Müller et al., 2011; Holt et al., 2014; Yang et al., 2019). Pigs are the natural host of PRV, and PRV infection in pigs can cause nervous system disorders, respiratory diseases, abortion of pregnant sows, and piglet death, causing huge economic losses to the pig industry (Cui et al., 2018; He et al., 2019). Until 2011, the Bartha-K6 strain vaccine was widely used in China to control PR. PRV variants began to circulate in China at the end of 2011, and new features emerged after the virus-infected pigs (Yu et al., 2014; Wu et al., 2017). Many large-scale pig farms immunized with the Bartha-K/61 strain vaccine showed the epidemic of PRV (Cui et al., 2018; Sun et al., 2018). Phylogenetic analysis classified the mutants into PRV II type (Ye et al., 2015). To prevent and control the epidemic of PRV scientifically, it is necessary to understand the pathogenic mechanism of PRV mutants and analyze the changes in various biological signal pathways in host cells after PRV infection.

With the development of high-throughput sequencing technology, it is possible to transfer the study of virus–host cell interaction from the detailed decomposition to the whole system. By integrating bioinformatic data, a comprehensive understanding of viral infections can be achieved. Among these, transcriptomics, as a useful tool for the systematic study of the physiological and chemical states of cells, has emerged as an important tool for the study of the cellular mechanisms and molecular functions of viral infection (Zhang et al., 2017; Chen et al., 2022). The bioinformatics analysis of differentially expressed genes (DEGs) extracted from transcripts after virus infection is helpful for a comprehensive understanding of the host response (Ai et al., 2021). For example, the changes in various biological processes and related molecules in the core pathway provide an important basis for the analysis of viral pathogenesis (Zhang et al., 2017; Liu et al., 2018; Reyes et al., 2018). At present, there are few reports about the transcriptomic differences of PK15 cells infected by PRV with different virulence. The Bartha-K/61 vaccine appeared to provide only suboptimal protection against these variants (Wu et al., 2013; Sun et al., 2018), although other studies do show adequate protection against such variants (An et al., 2013; Wang and Zhang, 2019). Because of this controversy, we attempted to analyze the differences between the PRV variant and conventional vaccine strain on infected host cells by transcriptomic techniques. In this study, the transcripts of PK15 cells infected with the PRV SD2017 variant strain and Bartha-K/61 strain were analyzed. The results provide a reference for understanding the mechanism of pathogenicity and immune evasion of the PRV mutants.

## Materials and methods

### Virus and cell lines

The wild-type PRV mutant SD2017 strain was isolated from the brain of a PRV-infected piglet in December 2017 in Linyi, Shandong province. Sequence analysis showed that both the 48th and 497th amino acid sequences of gE protein had L-Aspartic Acid insertion (D), which was consistent with the mutation characteristics of PRV type II mutants prevalent in China. The SD2017 strain was preserved in the Chinese General Microbiological Culture Collection Center (No. 22047). The main genomic information of the SD2017 strain has been published in GenBank (Acc. No. MW535259-MW535265). The PRV Bartha-K/61 vaccine strain was obtained from Shandong Huahong Biological Engineering Co., Ltd. PK15 (Sus scrofa epithelial kidney) cells used for PRV culture were obtained from the American Type Culture Collection (Manassas, VA, USA).

### Cell culture and virus infection

PK15 porcine kidney cells were cultured in DMEM (Gibco, Grand Island, NY, USA) containing 10% fetal bovine serum (Gibco, Grand Island, NY, USA) at 37°C in 5% CO_2_ in a humidified incubator. Confluent PK15 cell monolayers were dispersed with 0.25% trypsin and 0.02% EDTA, seeded in 6 cm cell culture flasks, cultured for 24 h to 70% confluency, and washed two times with PBS before virus infection. PRV SD2017 and PRV Bartha-K/61 were added at an MOI of 0.1 for 1 h, and the cells were then washed followed by the addition of 2% FBS/DMEM. PBS was used for mock-infected control. Cells were harvested at 24 h post-infection (hpi) in three independent biological replicates. A porcine pseudorabies virus (gB gene) Real-time PCR Detection Kit (Biotephy, Qingdao, China) was used for the quantitative detection of PRV.

Total RNA from PRV SD2017 strain-infected, Bartha-K/61 strain-infected, and non-infected PK15 cells was extracted using TRIzol reagent (Invitrogen, Shanghai, China), and the concentration and purity of RNA samples were determined using a NanoDrop ND-1000 spectrophotometer (Nano Drop Inc., Wilmington, DE, USA). The integrity of total RNA samples was determined using an Agilent 2100 Bioanalyzer system (Agilent Technologies, Santa Clara, CA, USA).

### Library construction and transcriptome sequencing

A total amount of 1 μg RNA per sample was used as input material for the RNA sample preparations. Sequencing libraries were generated using a NEBNext^®^ UltraTM RNA Library Prep Kit for Illumina^®^ (NEB, USA), following the manufacturer's recommendations, and index codes were added to attribute sequences to each sample.

To preferentially select cDNA fragments of 250–300 bp in length, the library fragments were purified with the AMPure XP system (Beckman Coulter, Beverly, USA). Then, 3 μl of USER Enzyme (NEB, USA) was used with size-selected, adaptor-ligated cDNA at 37°C for 15 min, followed by 5 min at 95°C before PCR. Then, PCR was performed with Phusion High-Fidelity DNA polymerase, Universal PCR primers, and Index (X) Primer. At last, PCR products were purified (AMPure XP system), and library quality was assessed on an Agilent Bioanalyzer 2100 system. The clustering of the index-coded samples was performed on a cBot Cluster Generation System using a TruSeq PE Cluster Kit v3-cBot-HS (Illumina), according to the manufacturer's instructions. After cluster generation, the library preparations were sequenced on an Illumina NovaSeq platform, and 150 bp paired-end reads were generated.

### Data analysis

Raw data (raw reads) of FASTQ format were first processed through in-house Perl scripts. Reference genome and gene model annotation files Sus scrofa 11.1 were downloaded from Ensembl (ftp://ftp.ensembl.org/pub/release-91/fasta/sus_scrofa/dna) directly. The index of the reference genome was built using Hisat2 v2.0.5, and paired-end clean reads were aligned to the reference genome using Hisat2 v2.0.5. Feature Counts v1.5.0-p3 was used to count the read numbers mapped to each gene, and then, FPKM of each gene was calculated based on the length of the gene and reads count mapped to this gene. Differential expression analysis of two groups was performed using the DESeq2 R package (1.16.1). DESeq2 provides statistical routines for determining differential expression in digital gene expression data using a model based on the negative binomial distribution. The resulting *P*-values were adjusted using Benjamini and Hochberg's approach for controlling the false discovery rate. Genes with an adjusted *P*-value of <0.05 found by DESeq2 were assigned as differentially expressed. Gene Ontology (GO) enrichment analysis of DEGs was implemented by the cluster Profiler R package, in which gene length bias was corrected. GO terms with a corrected *P*-value of <0.05 were considered to be significantly enriched by differentially expressed genes. KEGG (http://www.genome.jp/kegg/) is a database resource for understanding high-level functions and utilities of the biological system. We used the cluster Profiler R package to test the statistical enrichment of differentially expressed genes in KEGG pathways.

### RT-qPCR validation of differentially transcribed genes

A total of nine genes with increased or decreased transcription levels were randomly selected according to the sequencing results, and glyceraldehyde-3-phosphate dehydrogenase (GAPDH) was used as the internal reference gene to validate the high-throughput sequencing results. Primers were designed using Premier 6.0 (Table 1). The total RNA of samples was reverse-transcribed into cDNA using HiScript^®^ II Q RT SuperMix for qPCR (Vazyme, Nanjing, China) as a template for qPCR. The relative transcription levels of each gene were calculated using the 2^−ΔΔCt^ method, and the *t*-test was performed using GraphPad Prism 5.0.

**Table 1:** Primers for real-time PCR.

**Gene name**	**Primer**	**Primer sequence (5′ → 3′)**	**Product size (bp)**
FKBP5	F	AGTCTCCCCAAAATTCCCTCG	148
	R	TCGCTCCTTCGTTGGGATTTG	
ARHGAP2	F	CTGCCCAACGGCTATGTGAC	73
	R	TGTTGTGCTGGCCCGACTCTC	
DUSP6	F	CAGCGACTGGAACGAGAATAC	123
	R	AACTCGGCTTGGAACTTACTG	
TCF19	F	GTTGGCAGAACTGGACGATGAG	132
	R	CGAGGACGCCCACGACGATT	
AIMP1	F	AAGTGCTGACTCAAAGCCTGTT	256
	R	CATTACCATTGCCTGAGATACTACT	
NPM1	F	GAAAAAACTCCTAAAACACCG	176
	R	CACTGCCAGAGATCTTGAAT	
VAPB	F	AACCCGACAGACCGGAATGTG	138
	R	ATCATAATCGAAAGGCTGTAACATC	
SLC25A40	F	ACGTTTCCAGGGAACGCTG	97
	R	TGCCATCACTAAGGTAGGAGG	
SLC37A4	F	GGACTCCGCAACCTGGACCCT	145
	R	AGCACGTCTTTACGCCAAATACC	

## Results

### Kinetics of PRV propagation in PK15 cells

**Figure 1:**
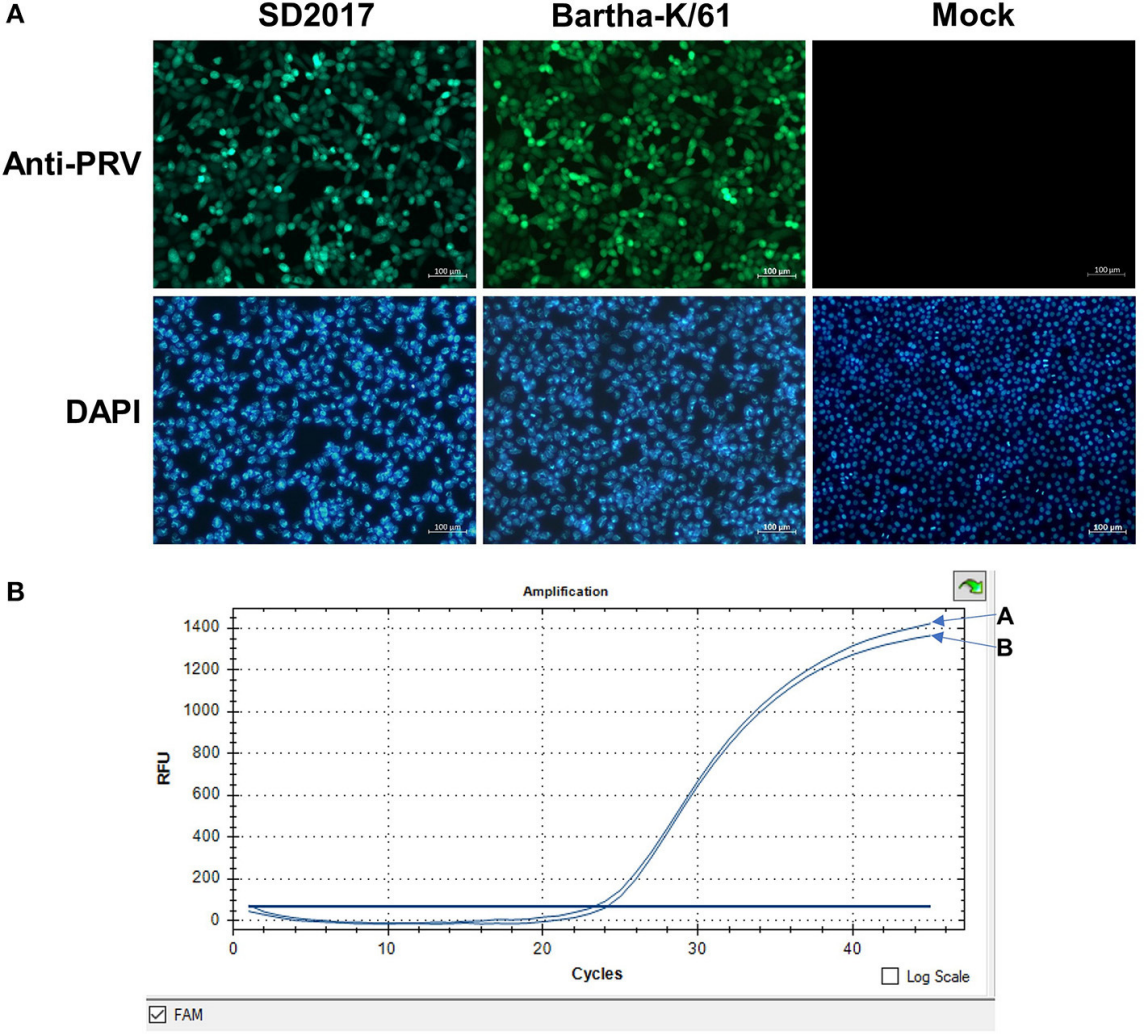
Detection of PRV in PK15 cells by fluorescence quantitative PCR. **(A)** PRV SD2017-infected PK15 cells. **(B)** PRV Bartha-K/61-infected PK15 cells.

PK15 cells were infected with the PRV SD2017 strain and Bartha-K/61 strain at 0.1 MOI, respectively. Quantitative analysis of PRV at 24 hpi was performed using a fluorescence quantitative PCR assay. CT values (23.8 and 24.2) showed that viral replication remained at a high level in both PRV-infected groups (Figure 1). Therefore, PK15 cells can be sampled for transcriptome sequencing at 24 hpi.

### Quality control of sequencing data

**Figure 2:**
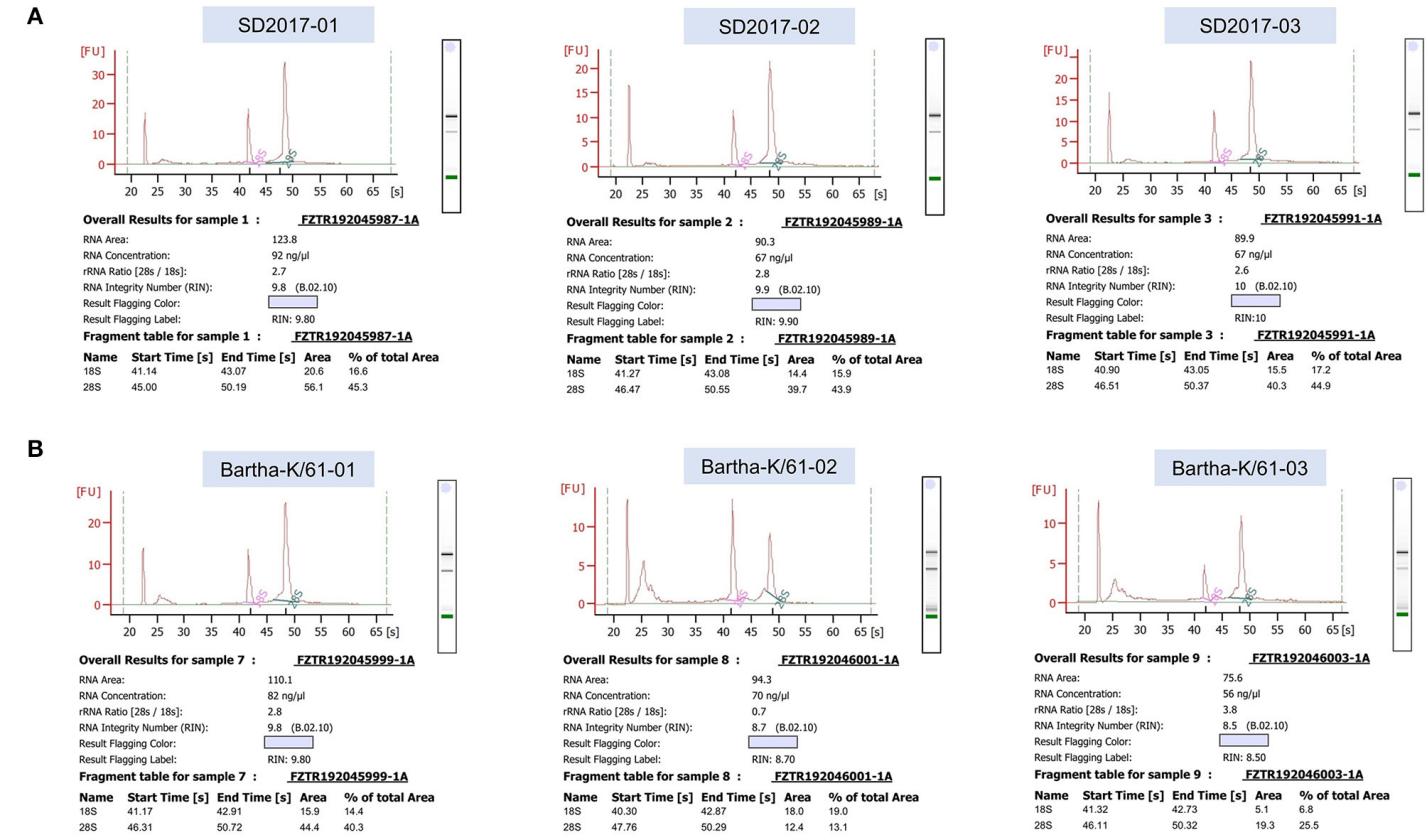
Results of RNA integrity testing of transcriptome sequencing samples. **(A)** PRV SD2017-infected PK15 cell samples. **(B)** PRV Bartha-K/61-infected PK15 cell samples.

RNA was extracted from PRV SD2017-infected PK15 cells and Bartha-K/61-infected PK15 cells (three replicates per group). RNA integrity was assessed using an Agilent 2100 bioanalyzer (Figure 2). The library was constructed according to the instructions of the NEBNext^®^ Ultra^TM^ RNA Library Prep Kit and sequenced using an Illumina high-throughput sequencing platform (Hi Seq/Mi Seq). The mean original readings for samples from the SD2017 (SD) group were 55,275,026 and 63,529,356 for samples from the Bartha-K/61 (BK) group. After filtering out low-quality reads, we obtained an average of 53,796,261 clean reads from the SD2017 group and 61,989,240 clean reads from the Bartha-K/61 group. Percentage values of Q20 and Q30 were higher than 97.71 and 94.03%, respectively (Table 2), which met data quality requirements and could be used for subsequent analysis.

**Table 2:** Analysis of RNA-seq sequencing data quality assessment.

**Sample**	**Raw_reads**	**Clean_reads**	**Clean_bases**	**Error_rate**	**Q20 (%)**	**Q30 (%)**	**GC (%)**
SD01	54,592,364	52,915,068	7.94G	0.02	98.12	94.60	56.20
SD02	53,995,846	52,509,870	7.88G	0.02	98.01	94.35	56.06
SD03	57,236,868	55,963,846	8.39G	0.03	97.90	94.03	56.03
BK01	63,211,510	61,728,968	9.26G	0.03	97.71	94.07	69.69
BK02	62,468,336	60,805,442	9.12G	0.02	97.87	94.38	68.95
BK03	64,908,222	63,433,310	9.51G	0.03	97.78	94.16	70.75

### Analysis of DEGs

**Figure 3:**
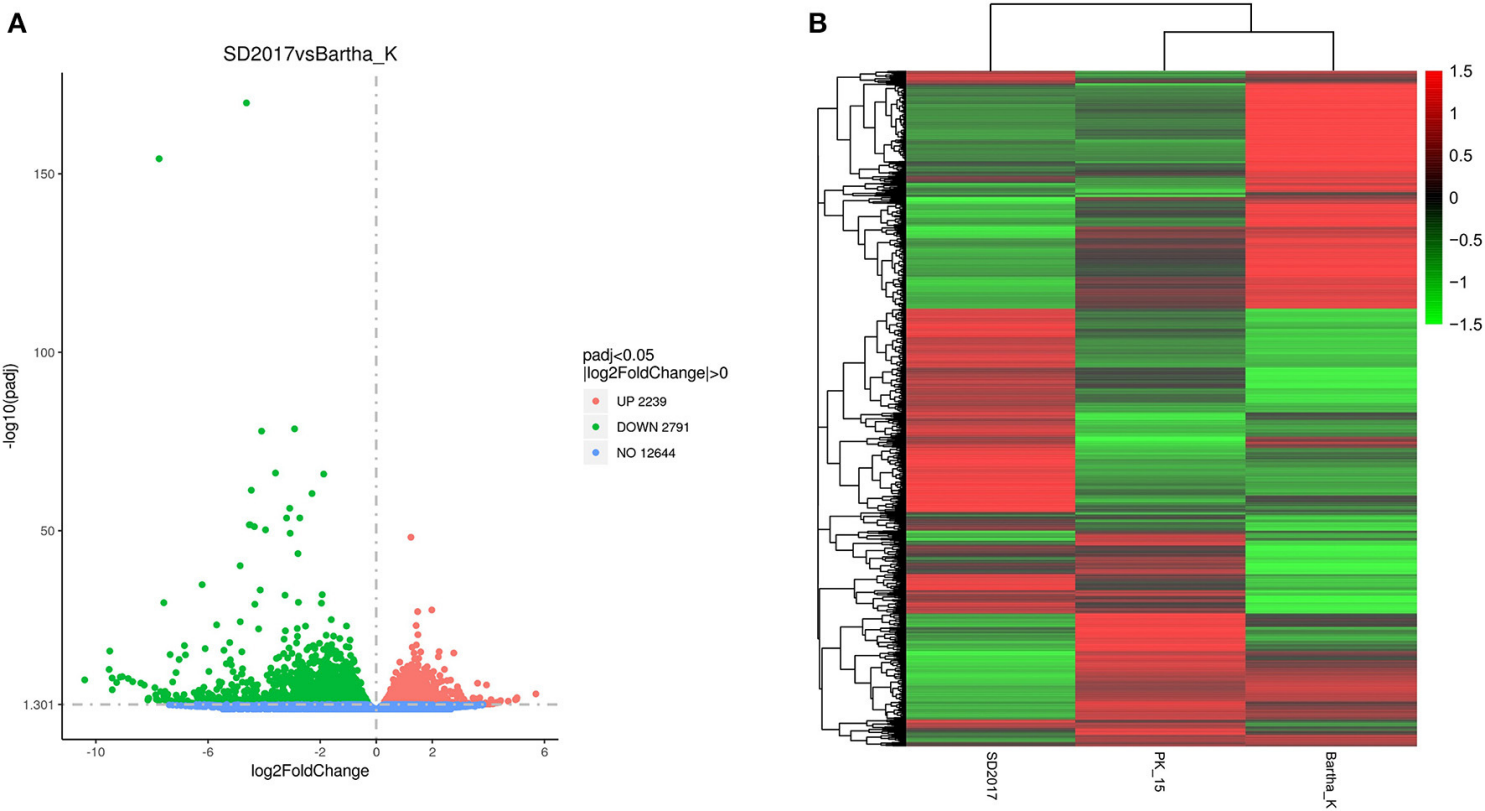
Quantitative analysis of DEGs. **(A)** Volcano plots of the distribution of DEGs. The *x*-axis is the log2FoldChange value, the *y*-axis is –log10padj, and the blue dotted line represents the threshold line of the differential gene screening criteria. **(B)** Cluster map of DEGs. The *x*-axis is the sample name, and the *y*-axis is the normalized value of the differential gene FPKM. The redder the color, the higher the expression, and the greener the color, the lower the expression.

To screen the DEGs of PRV SD2017 and Bartha-K/61-infected PK15 cells, DEG analysis was performed by DESeq2, as biological replicas were available in this study. Compared with Bartha-K/61 samples in |log2(FoldChange)| > 0 & padj < 0.05. A total of 2,239 significantly upregulated genes and 2,791 significantly downregulated genes were screened under the 0.05 criterion (Figure 3A; Supplementary Table S1). We used mainstream hierarchical clustering to perform cluster analysis on FPKM (fragments per kilobase million) values of genes and conducted Z-score for row homogenization. As shown in Figure 3B, different gene expression trends were observed in SD2017 and Bartha-K/61 samples, indicating that infection with these two strains induced significant gene expression changes in PK15 cells.

### GO Analysis of DEGs

**Figure 4:**
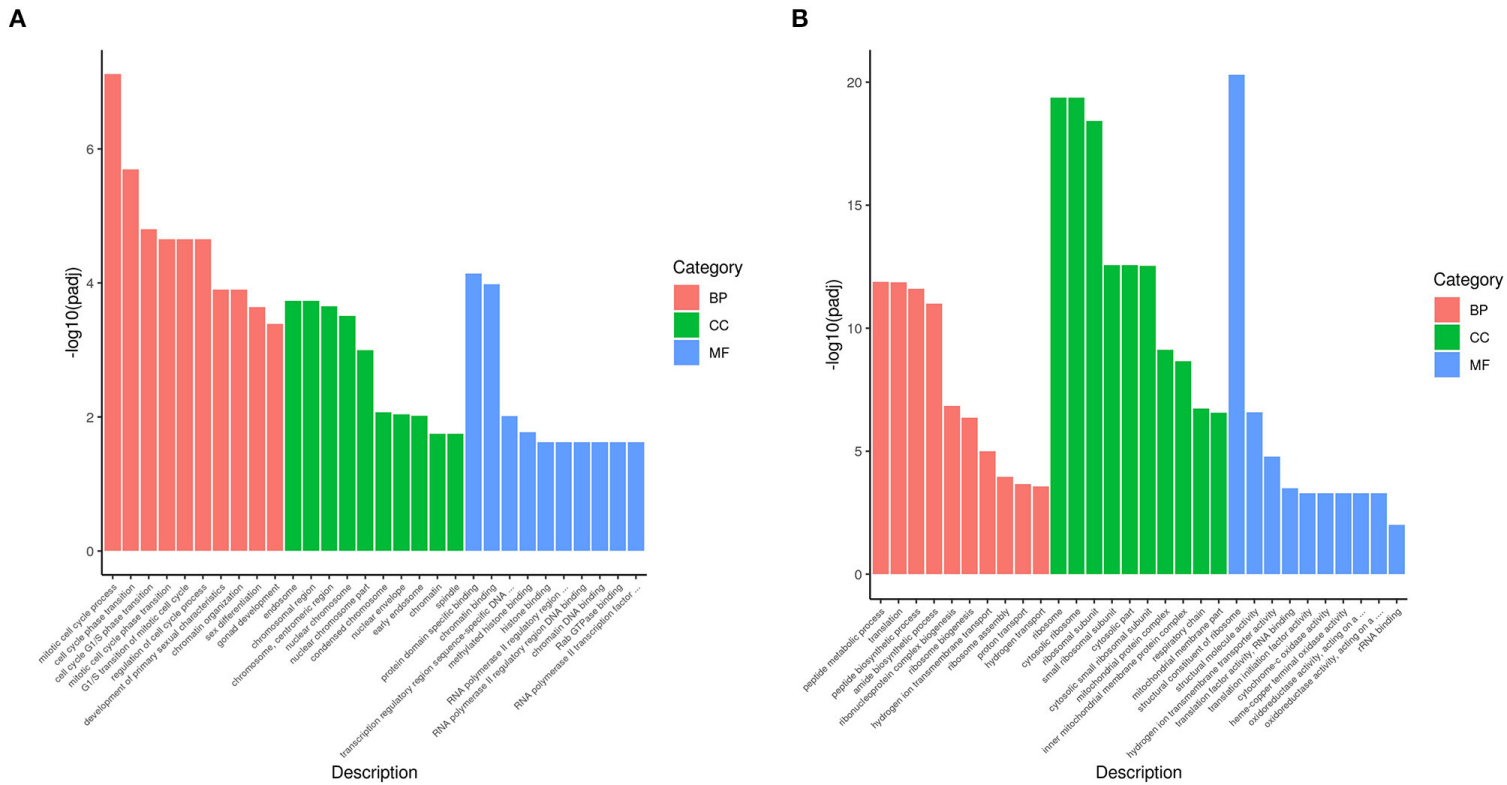
GO annotation of analysis of DEGs. **(A)** GO functional classification of the upregulated DEGs. **(B)** GO functional classification of the downregulated DEGs. The *x*-axis is GO Term. The *y*-axis is the significance level of GO term enrichment. The higher the value, the higher the significance. The different colors represent the three GO subclasses of BP, CC, and MF.

Gene Ontology (GO) is a comprehensive database describing gene function, which can be divided into three parts as follows: biological process, cellular component, and molecular function. Taking padj <0.05 as the threshold for significant GO enrichment, enrichment analysis results of DEGs GO in PRV SD2017 and Bartha-K/61-infected PK15 cells were obtained from Supplementary Table S2. The 30 terms with the most significant upregulation and downregulation were selected to draw bar charts for display (Figure 3). Among GO terms with significant enrichment of upregulated genes (Figure 4A), mitotic cell cycle process (GO:1903047), cell cycle phase transition (GO:0044770), cell cycle G1/S phase transition (GO:0044843), and mitotic cell cycle phase transition (GO:0044772) were the four most prominent in the BP category. In addition, protein domain-specific binding (GO:0019904) and chromatin binding (GO:0003682) were the most prominent GO terms in the MF category. In terms of the significantly enriched GO of downregulated genes (Figure 4B), ribosome (0042254), cytosolic ribosome (GO:0022626), and ribosomal subunit (GO:0044391) were the three most prominent GO terms in the CC category. Moreover, the structural constituent of ribosome (GO:0003735) was the most prominent GO term in the MF category. GO results showed that compared with Bartha-K/61 infection, SD2017 infection significantly upregulated DEGs enrichment mainly in the cell cycle, protein, and chromatin binding while downregulated DEGs were mainly enriched in ribosomes.

### KEGG analysis of DEGs

**Figure 5:**
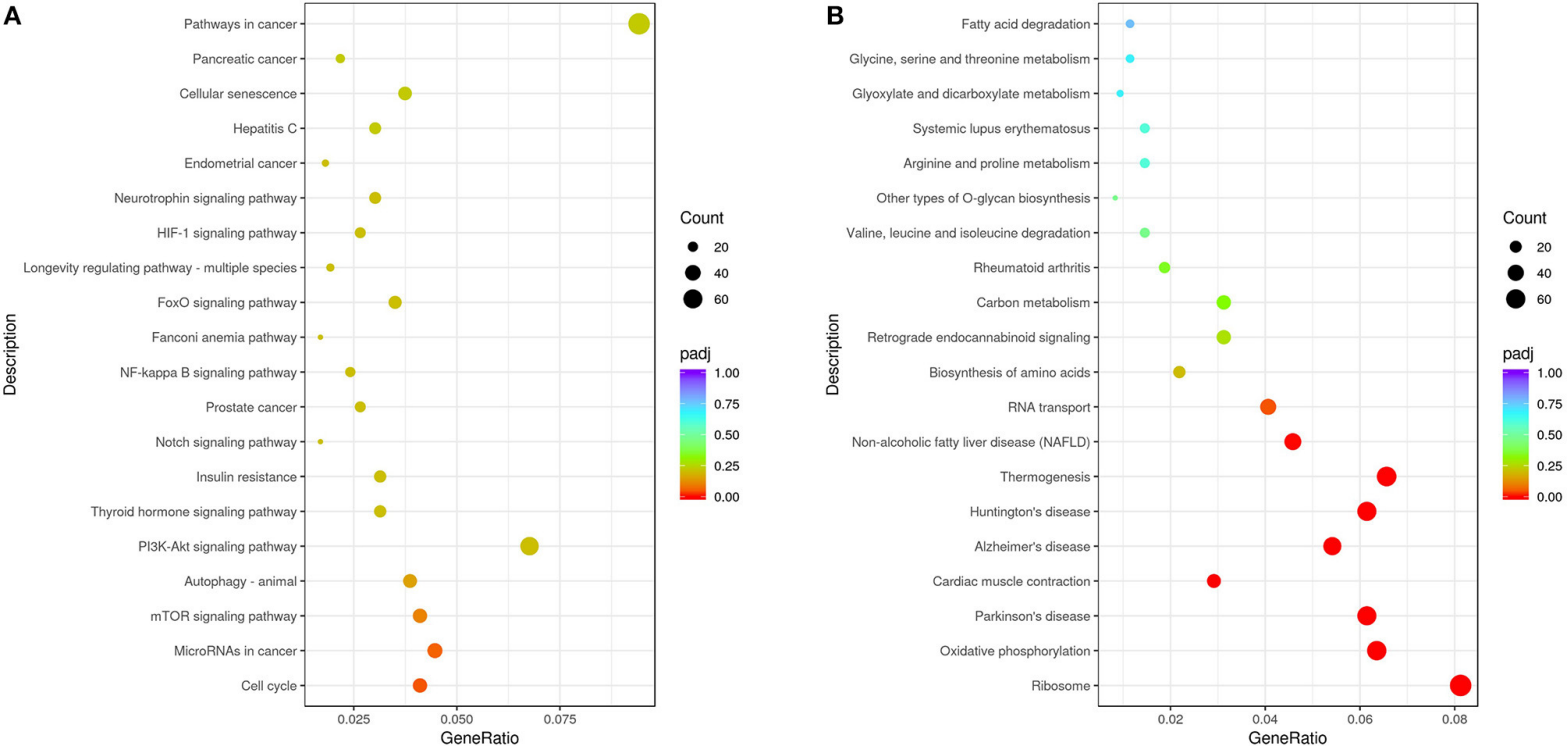
Analysis of KEGG enrichment. **(A)** Analysis of KEGG enrichment of the upregulated DEGs. **(B)** Analysis of KEGG enrichment of the downregulated DEGs. The *x*-axis is the ratio of the number of differential genes annotated to the KEGG pathway to the total number of differential genes. The *y*-axis is the KEGG pathway. The size of the dots represents the number of genes annotated to the KEGG pathway, and the color from red to purple represents the significance of the enrichment.

From the KEGG enrichment results (Supplementary Table S3), the most significant 20 KEGG pathways were selected to draw scatter plots for presentation, as shown in Figure 5. The KEGG enrichment analysis showed that pathways with the most upregulated DEGs enrichment (Figure 5A) were pathways in cancer (KEGG: ssc05200), cell cycle (KEGG: ssc04110), microRNAs in cancer (KEGG: ssc05206), mTOR signaling pathway (KEGG: ssc04150), and autophagy-animal (KEGG: ssc04140). The pathways with the most downregulated DEG enrichment (Figure 5B) were ribosome (KEGG: ssc03010), oxidative phosphorylation (KEGG: ssc00190), thermogenesis (KEGG: KEGG: ssc04714), Parkinson's disease (KEGG: ssc05012), and Alzheimer's disease (KEGG: ssc05010). These KEGG pathways were mainly related to cell cycle, signal transduction, and autophagy and were involved in virus–host cell interactions.

### Validation of the expression of DEGs by qRT-PCR

**Figure 6:**
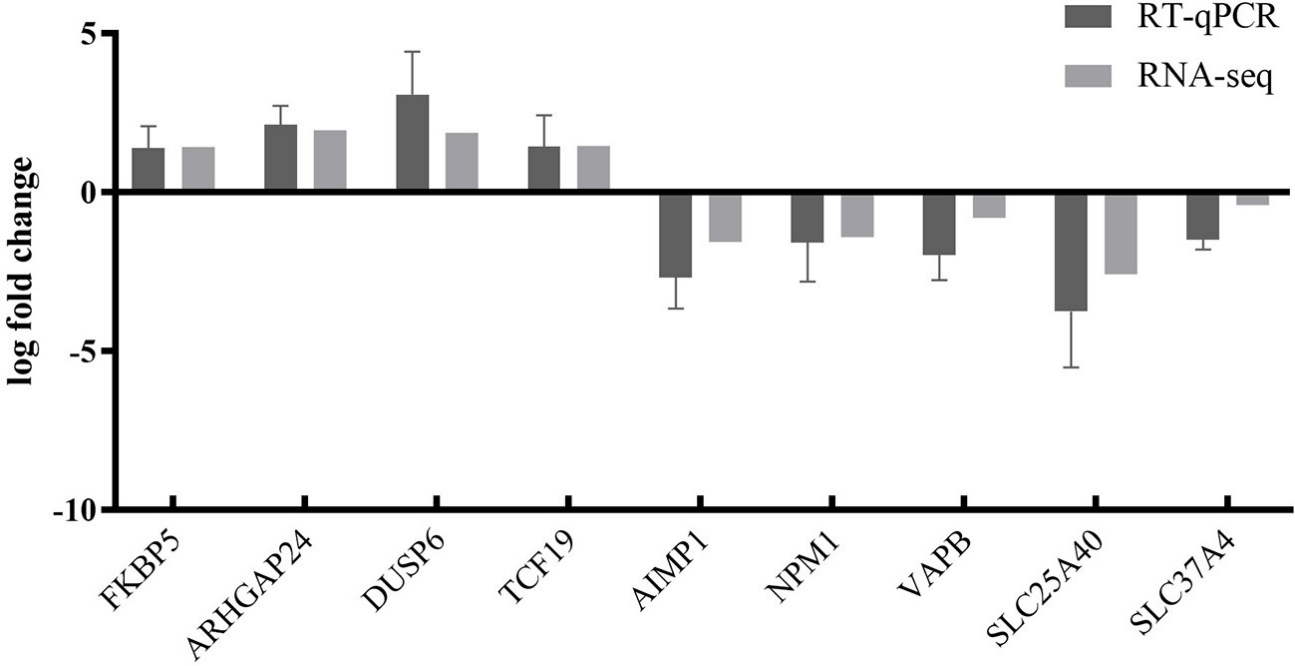
Comparison of fold changes of DEGs between RNA-seq and qRT-PCR. PK15 cells were infected with PRV SD2017 and Bartha-K/61 for 24 h; then, qRT-PCR was performed to detect the relative expression of selected DEGs. The horizontal axis represents the name, and the vertical axis indicates log2 fold changes of DEGs.

To further validate the transcriptome analysis results, we performed a qPCR analysis to determine the reproducibility of the differential gene expression. GAPDH mRNA was amplified as the endogenous control. A total of four upregulated genes (*FKBP5, ARHGAP24, DUSP6*, and *TCF19*) and five downregulated genes (*AIMP1, NPM1, VAPB, SLC25A40*, and *SLC37A4*) were analyzed. As shown in Figure 6 and Supplementary Table S4, the qRT-PCR results corresponded with transcriptome analysis results. Interestingly, many DEG expressed proteins are important regulators of host immune responses. For example, the upregulation of dual-specificity phosphatase 6 (DUSP6) impairs infectious bronchitis virus replication by negatively regulating the ERK pathway and promoting apoptosis (Ma C. et al., 2022). Aminoacyl tRNA synthetase complex interacting multifunctional protein 1 (AIMP1) regulates TCR signaling and induces differentiation of regulatory T cells by interfering with lipid raft binding (Chen et al., 2021). AIMp1 enhances Th1 polarization and is essential for effective antitumor and antiviral immunity (Liang et al., 2017). The absence of vesicle-associated membrane protein-associated protein B (VAPB) regulates autophagy in a Beclin 1-dependent manner (Escande-Beillard et al., 2020).

## Discussion

In recent years, high-throughput sequencing has been widely used in the study of differential transcriptomes caused by a viral infection, providing basic data for the analysis of viral infection mechanisms (Wang et al., 2019a; Ai et al., 2021). Recent reports have shown that functional lncRNAs and differential circRNA in PRV type II infected cells (Thomas et al., 2012; Rodríguez-Galán et al., 2021). However, there are few reports on the transcriptomic differences of PK15 cells infected by PRV with different virulence. Liu et al. analyzed the differential expression of miRNA induced by the PRV Fa ΔgE/gI strain and Fa wild strain in PK15 cells. GO analysis showed that the differentially expressed miRNA target genes in PK15 cells infected by PRV Faδge/gI and Fa wild strains were mainly involved in biological regulation and metabolic processes. STRING analysis showed that immune-related target genes of differentially expressed miRNAs in the toll-like receptor, B-cell receptor, T-cell receptor, nuclear factor-κB, and transforming growth factor-β signaling pathways were correlated (Wang et al., 2017). To comprehensively understand the changes in the total transcription level of cells infected with PRV type II mutant wild strain and traditional vaccine strain, the DEGs of PRV SD2017-infected PK15 cells and Bartha-K/61-infected PK15 cells were analyzed in this study. A total of 2,239 genes were upregulated. The expression of 2,791 genes was downregulated. These DEGs widely exist in cellular components, such as cell membranes and cytoplasm, and are involved in various intracellular processes. The results showed that the SD2017 infection caused a violent cell response. To identify the signaling pathways involved in DEGs, GO, and KEGG databases were used for enrichment analysis. GO functional annotation of differential genes showed that they were mainly enriched in cell cycle, protein and chromatin binding, and ribosome (Figure 4). A total of 13 KEGG pathways were significantly enriched, including cell cycle, mTOR signaling pathway, autophagy-animal, ribosome, oxidative phosphorylation, thermogenesis, Parkinson's disease, and Alzheimer's disease (Figure 5). These results suggest that PRV type II mutant wild strain infection has extensive effects on host cells. This is the first report of differential transcriptome infecting pig cell lines with PRV mutant wild strain and Bartha-K/61 vaccine strain.

Previous studies have shown that PRV infection can lead to changes in cellular immunity, metabolism, nucleic acid degradation, biosynthesis, MAPK, and many other biological processes and pathways. For example, PRV-encoded UL13 protein kinase acts as an antagonist of innate immunity by targeting IRF3 signaling pathways (Lv et al., 2020). PRV mediates apoptosis and DNA degradation by inducing oxidative stress and MAPK pathways (Yeh et al., 2008; Lai et al., 2019). Heat shock protein 27 (Hsp27) attenuates cGAS-mediated IFN-β signaling through ubiquitination of cGAS and promotes PRV infection (Li et al., 2022). PRV infection can induce the degradation of interferon type I receptors and lead to the upregulation of interferon-stimulated gene 15 (ISG15) expression (Zhang et al., 2017; Liu et al., 2018). The latency-associated transcript (LAT) gene is the only transcriptional region during latent infection of PRV that plays the key role in regulating viral latent infection and inhibiting apoptosis (Deng et al., 2022). The GO and KEGG enrichment results of DEGs in this study are consistent with the above conclusions. Compared with Bartha-K/61, PRV SD2017-infected PK15 cells, aminoacyl tRNA synthetase complex interacting multifunctional protein 1 (AIMP1), transforming growth factor beta induced (TGFBI), and tetraspanin CD9 were downregulated. In particular, CD9 is a key regulator of cell adhesion in the immune system (Reyes et al., 2018). These results suggest that the immune response of host cells to PRV SD2017 infection may be mediated by the above immune-related pathways. In addition, the peptide metabolic process, amide biosynthetic process, ribonucleoprotein complex biogenesis, ribosome assembly, mitochondrial protein complex, and other metabolism-related pathways have been enriched. This has suggested that PRV SD2017 infection may break the original material metabolism and biosynthesis process of PK15 cells, which is significantly different from Bartha-K/61-infected cells. In conclusion, the differential transcriptome data caused by infection of PRV variants in this study are the basis for analyzing the interaction between virus and host cells at the molecular level and also the premise for further exploring the pathogenic mechanism and immune response of PRV variants.

Viruses use a variety of strategies and molecular targets to influence host cell processes. These include cell cycle regulation, cytokine-mediated signaling, and immune response. Moreover, viruses often manipulate the host cell cycle to create a favorable environment for replication (Fan et al., 2018). It has been reported that RIPK3-dependent necroptosis limits PRV replication in PK15 cells (Gou et al., 2021). Although there have been many reports on the pathogenesis of PRV and its interaction with the host in recent years, the changes in the target cells of PRV after infection remain unclear (Li et al., 2019; Wang et al., 2019b). Our results showed that infection of PRV SD2017 significantly upregulated the cell cycle pathway of PK15 cells compared with Bartha-K/61. The related DEGs in the cell cycle pathway include ORC1, CDKN1B, SMAD3, CDC25C, MCM7, FZR1, CDC23, CDC25B, and CDC14B. The expression of these DEGs affects the cell cycle and thus the replication of the virus. Especially, short protein-binding motifs in ORC1 and CDC6 control the initiation of DNA replication (Hossain et al., 2021). Cyclin-dependent kinase inhibitor 1B (CDKN1B) mediates apoptosis of neuronal cells and inflammation induced by oxyhemoglobin via miR-502-5p (Chen et al., 2020). As an important cell cycle regulatory protein, cell division cycle 25C (CDC25C) activates the cyclin B1/CDK1 complex in cells for entering mitosis and regulates G2/M progression (Liu et al., 2020). Fizzy-related 1 (FZR1) is an activator of the anaphase-promoting complex/cyclosome (APC/C) and an important regulator of the mitotic cell division cycle (Holt et al., 2014). Cell division cycle 25 B (CDC25B) is a member of the CDC25 phosphatase family. It can dephosphorylate cyclin-dependent kinases and regulate the cell division cycle. Moreover, siRNA knockdown of CDC25B impairs influenza A virus (IAV) replication (Cui et al., 2018). Cell division cycle 14B (CDC14B) regulates mammalian RNA polymerase II and represses cell cycle transcription (Guillamot et al., 2011). The findings of these DEGs help us to understand the mechanism of PRV variant strain infection affecting host cells and provide new ideas for the development of targeted drugs.

At present, there have been many studies on the interaction between PRV and host natural immune signaling pathways. For example, after PRV infects cells, TNF-α can induce autophagy by activating p38 MAPK and JNK/SAPK signaling pathways (Yeh et al., 2008). In addition, PRV can degrade JAK through the proteasome pathway and inhibit the expression of interferon-stimulating genes (Yin et al., 2021). Compared with Bartha-K/61-infected cells, it was also found that PRV SD2017-infected PK15 cells showed DEGs in multiple innate immune pathways, such as the mTOR signaling pathway, autophagy-animal, the NF-κB signaling pathway, the TNF signaling pathway, and the NOD-like receptor signaling pathway. Autophagy plays a crucial role in maintaining cellular homeostasis and is closely related to the occurrence of a variety of diseases. Many studies have shown that a number of signal transduction pathways are involved in the regulation of autophagy (Jung et al., 2010; Yu et al., 2010; Wang and Zhang, 2019). Previous research has shown that the tegument protein UL21 (unique long region 21) in PRV dampens type I interferon signaling by triggering the degradation of CGAS (cyclic GMP–AMP synthase) through the macroautophagy/autophagy–lysosome pathway (Ma Z. et al., 2022). PRV induced autophagy via the classical Beclin-1-Atg7-Atg5 pathway to enhance viral replication in N2a cells *in vitro* (Xu et al., 2018). PRV infection triggers persistent NF-κB activation in an unorthodox way and dramatically modulates the NF-κB signaling axis, preventing typical proinflammatory gene expression and the responsiveness of cells to canonical NF-κB signaling, which may aid the virus in modulating early proinflammatory responses in the infected host (Romero et al., 2020). These results are helpful to further explore the molecular mechanism of the PRV variant escaping host immunity.

In conclusion, in this study, differential transcriptome data of PRV SD2017 and Bartha-K/61 strains-infected PK15 cells were obtained by high-throughput sequencing and bioinformatic analysis. We also enriched DEGs into various biological processes, such as metabolism, immunity, biosynthesis, cell cycle, autophagy, and NF-κB signaling pathways. It provided basic data for further study on the molecular mechanism of PRV variant infection.

## Data availability statement

The original contributions presented in the study are included in the article/Supplementary material, further inquiries can be directed to the corresponding authors.

## Author contributions

HZ and RZ designed the experiments, wrote the manuscript, and analyzed the data analysis. HZ, XD, SD, YL, and JL carried out the experiments. GL and XC performed writing—review and editing. XC and HS checked the manuscript. All authors read and approved the final version. All authors agree to be accountable for the content of the study.
